# Perceptions and attitudes toward sexual norms: key insights from the International Society of Sexual Medicine Young Researchers Committee survey

**DOI:** 10.1093/sexmed/qfaf032

**Published:** 2025-05-18

**Authors:** Bruno Nascimento, Edoardo Pozzi, Luca Boeri, Paolo Capogrosso, Helen Bernie, Petar Bajic, Alessandra Fisher, Borja Garcia-Gomez, Uros Milekovic, Carolyn Salter, Vaibhav Modgil, Hisanori Taniguchi, Filipe Tenorio, Ioannis Sokolakis, Andrea Salonia

**Affiliations:** Division of Urology, Hospital das Clinicas – University of Sao Paulo Medical School, Sao Paulo, SP 05403-010, Brazil; Vita-Salute San Raffaele University, Milan 20132, Italy; Division of Experimental Oncology/Unit of Urology, URI; IRCCS Ospedale San Raffaele, Milan 20132, Italy; Department of Urology, Foundation IRCCS Ca’ Granda – Ospedale Maggiore Policlinico, University of Milan, Milan 20122, Italy; Department of Urology, Circolo & Fondazione Macchi Hospital – ASST Sette Laghi, Varese 21100, Italy; IU Health Physicians Urology, Indianapolis, IN 46202, United States; Cleveland Clinic, Cleveland, OH 44195, United States; University of Florence, Firenze 50121, Italy; University Hospital October 12, Madrid 28040, Spain; KU Leuven, Leuven 3000, Belgium; Madigan Army Medical Center, Tacoma, WA 98431, United States; Manchester Royal Infirmary, Manchester, M13 9WL, United Kingdom; Kansai Medical University, Hirakata, Osaka 573-1010, Japan; IMIP Urology, Boa Vista 57000-000, Brazil; Julius Maximilian University of Würzburg, Würzburg, Bayern 47057, Germany; Vita-Salute San Raffaele University, Milan 20132, Italy; Division of Experimental Oncology/Unit of Urology, URI; IRCCS Ospedale San Raffaele, Milan 20132, Italy

**Keywords:** sexual norms, International Society of Sexual Medicine, Sexual Medicine, epidemiology

## Abstract

**Background:**

Sexuality is a complex aspect of human life, and the perception of sexual normality may vary across genders, religious beliefs, and other aspects.

**Aim:**

To report preliminary findings of a pilot survey on sexual attitudes, behaviors, and individual perception of sexual normality in a contemporary cross-cultural scenario.

**Methods:**

A 48-item survey was developed by the Young Researchers Committee (YRC) on behalf of the International Society for Sexual Medicine (ISSM) to collect data on cross-cultural perceptions and attitudes toward sexual norms. The survey consisted of questions related to sexual attraction, behavior, identity, orientation, and subjective perception of sexual normality. Data were collected via five translated versions across five countries (Italy, United States, Brazil, Spain, and Japan) and analyzed to investigate how cultural norms, personal experiences, and social expectations shape individuals’ views on sexual normality.

**Outcomes:**

The primary outcome was to assess gender-based differences in sexual behaviors, satisfaction, religious beliefs’ impact, pornography use, and anatomical perceptions.

**Results:**

This pilot study included 3423 respondents [63.5% female, 36.2% male; median (IQR) age 39 (30.00, 50.00) years]. Of all, active sexual life was reported by 83.3% participants, with 58.8% expressing satisfaction with their sex life. Heterosexual orientation was predominant (90%), with significant differences in distribution between genders in terms of sexual orientation (*P* < .001). Religious influence on sexual activity was reported by 18.6% of respondents, more commonly among females (20.2% vs 15.8%, *P* < .001). Median ages of first sexual intercourse and pornography exposure were 18 (16.00, 19.00) and 14 (12.00, 16.00) years, respectively, with females reporting older ages for both experiences (*P* < .001). Regarding perceptions of normality, most respondents (55.6%) believed first sexual intercourse typically occurs between 16 and 19 years. The perception of normal erect penile length differed between genders, with men more likely to report greater values (>16.1 cm: 13.1% vs 6.2%, *P* < .01). Gender differences were also observed in orgasm frequency, with fewer females reporting orgasm during >80% of sexual encounters (38.2% vs 66.5%, *P* < .001).

**Clinical Implications:**

Our findings shape the development of sexual education, fostering inclusivity, equity, and sexual health for overall satisfaction.

**Strengths and Limitations:**

Possible biases associated with different modalities throughout data collections and with different linguistic and cultural weights given the cross-sectional nature of the pilot survey.

**Conclusion:**

Current preliminary findings from the pilot survey developed by the ISSM YRC start shedding lights on perceptions and attitudes toward sexual norms, and gender differences in sexual behaviors and satisfaction.

## Introduction

Sexuality is a complex and intricate aspect of human life, encompassing a broad spectrum of sexual attraction, behaviors, identity, and orientation.^1^^,^^2^ The concept of sexual normality is determined by societal standards within a specific cultural context.^3^ However, the perception of what is considered “normal” greatly varies across different cultures, societies, and beliefs.^4^^,^^5^ As such, not only cultural backgrounds, age, and gender identity, but also the cultural level, the socio-cultural and religious environment, personal experiences, and exposure to different sources of information play a major role in shaping perceptions of sexual normality. These demographic and socio-cultural factors interact in complex ways, influencing individual attitudes and beliefs.^4^^,^^6^ For instance, some cultures, address sexual behavior outside of marriage as a taboo, while others are more likely to consider it as socially acceptable (in other terms, “normal”). In this regard, different cultures can have varying attitudes and beliefs about gender roles and expectations regarding sexual behaviors. Some cultures demonstrate greater acceptance of diverse sexual expressions and relationship structures, while others maintain more restrictive views about what constitutes appropriate sexual behavior.^4^^,^^6^ Additionally, social expectations influenced by media and/or religion,^7-9^ can establish rules about sexual behaviors, shaping again what is deemed acceptable or unacceptable.^4^ It is important to recognize that these expectations can be restrictive and limit individual freedom and expression.^8^

Therefore, the complex biopsychosocial factors influencing sexual practices, perceptions of normalcy, and distress are well recognized, yet the extent of their impact and the most relevant contributing factors remain largely understudied. Furthermore, recent societal changes—such as increased social media engagement and widespread pornography accessibility—may have significantly shape these dynamics. As such, a distorted perception of normality can potentially create pressure on an individual’s sexual self-perception. Sexual Medicine practitioners must acknowledge the influence of social expectations on their patients’ mental and physical well-being, ensuring a non-judgmental approach that allows individuals to freely express their sexual concerns while also refining clinicians’ ability to tailor treatment strategies. As part of an international collaboration effort to shed light to these rarely studied aspects, the Young Researchers Committee (YRC) on behalf of the International Society for Sexual Medicine (ISSM) developed a pilot survey regarding perception of sexual norms. Thereof, through a specifically developed questionnaire, the aim of this pilot multicenter international study was to explore how participant characteristics influence sexual attitudes, behaviors, satisfaction, and perceptions of sexual normality in a contemporary scenario.

## Materials and methods

### Study design and data collection

We conducted a multicenter international pilot survey using a mixed-mode data collection approach. Data were collected through a 48-item questionnaire developed by the ISSM YRC. The recruitment strategy encompassed three distinct methods, as follows: (1) on site, direct distribution to respondents at medical centers; (2) online distribution, through institutional emails; and (3) dissemination through institutional social media platforms.

### Survey development

Considering a bio-psycho-social conceptual model for the understanding of sexuality and through an iterative consensus process among members of the ISSM YRC, comprising urologists, psychologists, and sexologists, the questionnaire was designed to evaluate how socio-cultural norms, personal experiences, and social expectations influence individuals’ perceptions of sexual normality. All committee members proposed and reviewed questions through multiple rounds of revision. The final instrument comprised 48 items structured across four domains, Table 1. The first domain included 13 questions addressing demographics, followed by the second domain with 9 questions on sexual background. The third domain consisted of 5 questions regarding current sexual habits, and the fourth domain contained 21 questions examining personal perception of sexual normality according to gender assigned at birth.

**Table 1:** Overall cohort responses to the questionnaire and responses stratified by gender assigned as birth (female vs male) and according to sexual satisfaction (not satisfied vs satisfied).

**Question**	**Overall**	**Gender at birth (female)**	**Gender at birth (male)**	***P* value**	**Sexual satisfaction (not satisfied)**	**Sexual satisfaction (satisfied)**	***P* value**
**1. Age (year), Median [IQR]**	39.00 [30.00, 50.50]	39.00 [30.00, 50.00]	40.00 [30.00, 52.00]	.02	41.00 [31.00, 52.00]	38.00 [30.00, 49.00]	<.001
**2. What gender were you assigned at birth (such as on an original birth certificate)? (%)**				Ref			.06
Female	2175 (63.5)	2175 (100.0)	0 (0.0)		823 (65.8)	1243 (61.7)	
Male	1238 (36.2)	0 (0.0)	1238 (100.0)		426 (34.1)	764 (38.0)	
Missing	10 (0.3)	0 (0.0)	0 (0.0)		2 (0.2)	6 (0.3)	
**3. How do you experience your own gender? (%)**				<.001			.03
Both man and woman	10 (0.3)	6 (0.3)	4 (0.3)		6 (0.5)	3 (0.1)	
Man	1238 (36.2)	29 (1.3)	1209 (97.7)		429 (34.3)	763 (37.9)	
Neither	19 (0.6)	14 (0.6)	4 (0.3)		4 (0.3)	15 (0.7)	
Woman	2139 (62.5)	2119 (97.4)	16 (1.3)		808 (64.6)	1222 (60.7)	
Missing	17 (0.5)	7 (0.3)	5 (0.4)		4 (0.3)	10 (0.5)	
**4. Sexual orientation (%)**				<.001			.2
Asexual	13 (0.4)	12 (0.6)	1 (0.1)		6 (0.5)	7 (0.3)	
I am attracted toward both genders	11 (0.3)	4 (0.2)	6 (0.5)		5 (0.4)	5 (0.2)	
I am attracted toward men	2245 (65.6)	2056 (94.5)	185 (14.9)		844 (67.5)	1288 (64.0)	
I am attracted toward women	1089 (31.8)	52 (2.4)	1036 (83.7)		369 (29.5)	677 (33.6)	
I do not know	9 (0.3)	9 (0.4)	0 (0.0)		4 (0.3)	5 (0.2)	
Pansexual	46 (1.3)	37 (1.7)	8 (0.6)		21 (1.7)	24 (1.2)	
Missing	10 (0.3)	5 (0.2)	2 (0.2)		2 (0.2)	7 (0.3)	
**5. Relationship status (%)**				<.001			<.001
Divorced	198 (5.8)	154 (7.1)	43 (3.5)		111 (8.9)	81 (4.0)	
Married	1686 (49.3)	1065 (49.0)	619 (50.0)		530 (42.4)	1053 (52.3)	
Partnered	713 (20.8)	424 (19.5)	289 (23.3)		186 (14.9)	517 (25.7)	
Single	764 (22.3)	493 (22.7)	269 (21.8)		396 (31.7)	333 (16.5)	
Widowed	40 (1.2)	31 (1.4)	8 (0.6)		19 (1.5)	19 (0.9)	
Missing	22 (0.6)	8 (0.4)	10 (0.8)		9 (0.6)	10 (0.5)	
**6. Relationship duration (year), Median [IQR]**	10.00 [5.00, 20.00]	10.00 [5.00, 20.00]	10.00 [4.00, 20.00]	.9	12.00 [5.00, 20.00]	10.00 [5.00, 20.00]	.03
**7. Have you been sexually active in the last 12 months? (%)**				<.001			<.001
No	503 (14.7)	358 (16.5)	143 (11.6)		337 (26.9)	134 (6.7)	
Yes	2852 (83.3)	1771 (81.4)	1075 (86.8)		888 (71.0)	1851 (92.0)	
Missing	68 (2.0)	46 (2.1)	20 (1.6)		26 (2.1)	28 (1.4)	
**8. What is your religion? (%)**				<.001			.1
Agnosticism	21 (0.6)	6 (0.3)	15 (1.2)		9 (0.7)	12 (0.6)	
Atheist	8 (0.2)	1 (0.0)	7 (0.6)		6 (0.5)	2 (0.1)	
Buddhist	41 (1.2)	28 (1.3)	13 (1.1)		17 (1.4)	23 (1.1)	
Christian-Catholic	1350 (39.4)	817 (37.6)	531 (42.9)		479 (38.3)	837 (41.6)	
Christian-Orthodox	21 (0.6)	17 (0.8)	4 (0.3)		7 (0.6)	13 (0.6)	
Christian-Protestant	489 (14.3)	349 (16.0)	138 (11.1)		179 (14.3)	284 (14.1)	
Hindu	13 (0.4)	7 (0.3)	6 (0.5)		5 (0.4)	5 (0.2)	
Jewish	51 (1.5)	37 (1.7)	14 (1.1)		22 (1.8)	24 (1.2)	
Judaica	8 (0.2)	6 (0.3)	2 (0.2)		3 (0.2)	5 (0.2)	
Muslim	23 (0.7)	19 (0.9)	4 (0.3)		11 (0.9)	8 (0.4)	
None	1048 (30.6)	630 (29.0)	414 (33.4)		383 (30.6)	594 (29.5)	
Other	24 (0.7)	11 (0.5)	13 (1.1)		9 (0.7)	14 (0.7)	
Seventh-day Adventist Church	5 (0.1)	2 (0.1)	3 (0.2)		2 (0.2)	3 (0.1)	
Spiritism	186 (5.4)	145 (6.7)	41 (3.3)		76 (6.1)	109 (5.4)	
Taoism	1 (0.0)	0 (0.0)	1 (0.1)		0 (0.0)	1 (0.0)	
Umbanda	55 (1.6)	42 (1.9)	13 (1.1)		12 (1.0)	43 (2.1)	
Missing	79 (2.3)	58 (2.7)	19 (1.5)		31 (2.5)	36 (1.8)	
**9. What is your opinion regarding the following statement: my religious beliefs impact decisions regarding my sexual activity? (%)**				<.001			.016
Agree	638 (18.6)	440 (20.2)	196 (15.8)		240 (19.2)	375 (18.6)	
Disagree	1167 (34.1)	801 (36.8)	361 (29.2)		433 (34.6)	704 (35.0)	
Neutral	722 (21.1)	449 (20.6)	273 (22.1)		267 (21.3)	430 (21.4)	
Strongly agree	57 (1.7)	34 (2.6)	23 (1.8)		27 (2.2)	19 (0.9)	
Strongly disagree	763 (22.3)	404 (18.6)	358 (28.9)		252 (20.1)	453 (22.5)	
Missing	76 (2.2)	47 (2.2)	27 (2.2)		32 (2.6)	32 (1.6)	
**10. Which of the below best describe your highest completed level of education? (%)**				<.001			.008
College-University	1212 (35.4)	725 (33.3)	484 (39.1)		437 (34.9)	727 (36.1)	
Elementary-basic school	415 (12.1)	283 (13.0)	131 (10.6)		157 (12.5)	253 (12.6)	
Graduate school	147 (4.3)	62 (2.9)	85 (6.9)		36 (2.9)	110 (5.5)	
High-secondary school	266 (7.8)	119 (5.5)	147 (11.9)		106 (8.5)	144 (7.2)	
Post-graduate education	1325 (38.7)	942 (43.3)	379 (30.6)		494 (39.5)	755 (37.5)	
Missing	58 (1.7)	44 (2.0)	14 (1.0)		21 (1.7)	24 (1.1)	
**11. Are you currently employed or working for pay? (%)**				<.001			.5
No	363 (10.6)	190 (8.7)	173 (14.0)		138 (11.0)	203 (10.1)	
Yes	2989 (87.3)	1934 (88.9)	1047 (84.6)		1088 (87.0)	1777 (88.3)	
Missing	71 (2.1)	51 (2.3)	18 (1.5)		25 (2.0)	33 (1.6)	
**12. Number of children that you are responsible for? (%)**				<.001			.008
0	1628 (47.6)	999 (45.9)	627 (50.6)		589 (47.1)	990 (49.2)	
1	652 (19.0)	464 (21.3)	186 (15.0)		239 (19.1)	392 (19.5)	
2	639 (18.7)	400 (18.4)	235 (19.0)		231 (18.5)	393 (19.5)	
3	202 (5.9)	128 (5.9)	74 (6.0)		77 (6.2)	120 (6.0)	
4	26 (0.8)	18 (0.8)	8 (0.6)		7 (0.6)	17 (0.8)	
5	8 (0.2)	6 (0.3)	2 (0.2)		3 (0.2)	5 (0.2)	
6	8 (0.2)	8 (0.4)	0 (0.0)		5 (0.4)	3 (0.1)	
Missing	260 (7.6)	152 (7.0)	106 (8.6)		100 (8.0)	93 (4.6)	
**13. Age of the youngest children you are currently responsible for or helping raise (%)**				<.001			.04
11-15 years old	583 (17.0)	405 (18.6)	176 (14.2)		188 (15.0)	379 (18.8)	
16-20 years old	220 (6.4)	141 (6.5)	77 (6.2)		86 (6.9)	124 (6.2)	
5-10 years old	23 (0.7)	7 (0.3)	16 (1.3)		8 (0.6)	14 (0.7)	
>20 years old	431 (12.6)	277 (12.7)	153 (12.4)		179 (14.3)	240 (11.9)	
<5 years old	401 (11.7)	282 (13.0)	119 (9.6)		145 (11.6)	251 (12.5)	
Missing	1765 (51.6)	1063 (48.9)	697 (56.3)		645 (51.6)	1005 (49.9)	
**14. Approximate age of first pornography encounter (years), Median [IQR]**	14.00 [12.00, 16.00]	15.00 [12.00, 17.00]	13.00 [12.00, 15.00]	<.001	14.00 [12.00, 16.00]	14.00 [12.00, 16.00]	.4
**15. Approximate age of first sexual intercourse (years), Median [IQR]**	18.00 [16.00, 19.00]	18.00 [16.00, 20.00]	17.00 [16.00, 19.00]	.002	18.00 [16.00, 20.00]	17.00 [16.00, 19.00]	<.001
**16. Approximate number of lifetime sexual partners, Median [IQR]**	5.00 [3.00, 11.00]	5.00 [2.00, 10.00]	7.00 [3.00, 16.00]	<.001	5.00 [3.00, 10.00]	5.00 [3.00, 12.00]	.3
**17. Approximate number of current sexual partners, Median [IQR]**	1.00 [1.00, 1.00]	1.00 [1.00, 1.00]	1.00 [1.00, 1.00]	1	1.00 [0.00, 1.00]	1.00 [1.00, 1.00]	<.001
**18. Have you ever been coerced into sexual activity that you did not consent to? (%)**				<.001			<.001
No	2577 (75.3)	1527 (70.2)	1043 (84.2)		931 (74.4)	1629 (80.9)	
Yes	689 (20.1)	543 (25.0)	144 (11.6)		313 (25.0)	372 (18.5)	
Missing	157 (4.6)	105 (4.8)	51 (4.1)		7 (0.6)	12 (0.6)	
**19. Approximate number of lifetime sexual encounters involving a paid (commercial) sex worker? (%)**				<.001			.2
1-5	246 (7.2)	40 (1.8)	205 (16.6)		91 (7.3)	155 (7.7)	
11-20	29 (0.8)	5 (0.2)	24 (1.9)		11 (0.9)	18 (0.9)	
6-10	52 (1.5)	8 (0.4)	44 (3.6)		27 (2.2)	24 (1.2)	
> 20	48 (1.4)	13 (0.6)	35 (2.8)		22 (1.8)	26 (1.3)	
Never	2881 (84.2)	2006 (92.2)	868 (70.1)		1088 (87.0)	1777 (88.3)	
Missing	167 (4.9)	103 (4.7)	62 (5.0)		12 (1.0)	13 (0.6)	
**20. What is your opinion regarding the following statement: penetration is important to me during sexual encounter? (%)**				<.001			<.001
Agree	1292 (37.7)	796 (36.6)	495 (40.0)		464 (37.1)	823 (40.9)	
Disagree	334 (9.8)	257 (11.8)	77 (6.2)		142 (11.4)	192 (9.5)	
Neutral	599 (17.5)	384 (17.7)	209 (16.9)		281 (22.5)	312 (15.5)	
Strongly agree	944 (27.6)	565 (26.0)	378 (30.5)		314 (25.1)	626 (31.1)	
Strongly disagree	102 (3.0)	74 (3.4)	28 (2.3)		47 (3.8)	55 (2.7)	
Missing	152 (4.4)	99 (4.6)	51 (4.1)		3 (0.2)	5 (0.2)	
**21. How often do you have sexual fantasies that you find difficult to share with your partner? (%)**				.04			<.001
About Half the Time	445 (13.0)	265 (12.2)	180 (14.5)		196 (15.7)	247 (12.3)	
Always	158 (4.6)	99 (4.6)	59 (4.8)		105 (8.4)	53 (2.6)	
Never	616 (18.0)	406 (18.7)	208 (16.8)		189 (15.1)	426 (21.2)	
Seldom	1604 (46.9)	1041 (47.9)	558 (45.1)		507 (40.5)	1092 (54.2)	
Usually	422 (12.3)	247 (11.4)	175 (14.1)		237 (18.9)	185 (9.2)	
Missing	178 (5.2)	117 (5.4)	58 (4.7)		17 (1.4)	10 (0.5)	
**22. Overall, how satisfied are you with your sex life? (%)**				.007			
Dissatisfied	450 (13.1)	279 (12.8)	171 (13.8)		Ref	Ref	
Neither satisfied nor dissatisfied	710 (20.7)	486 (22.3)	222 (17.9)		Ref	Ref	
Satisfied	1358 (39.7)	8 (0.36)	532 (43.0)		Ref	Ref	
Very dissatisfied	91 (2.7)	58 (2.7)	33 (2.7)		Ref	Ref	
Very satisfied	655 (19.1)	422 (19.4)	232 (18.7)		Ref	Ref	
Missing	159 (4.6)	109 (5.0)	48 (3.9)		Ref	Ref	
**23. On average, how often do you use pornography? (%)**				<.001			<.001
Daily	181 (5.3)	43 (2.0)	138 (11.1)		96 (7.7)	85 (4.2)	
Monthly	834 (24.4)	513 (23.6)	321 (25.9)		292 (23.3)	539 (26.8)	
More than once a day	27 (0.8)	8 (0.4)	19 (1.5)		10 (0.8)	17 (0.8)	
Never	1577 (46.1)	1259 (57.9)	312 (25.2)		589 (47.1)	977 (48.5)	
Weekly	640 (18.7)	241 (11.1)	398 (32.1)		253 (20.2)	386 (19.2)	
Missing	164 (4.8)	111 (5.1)	50 (4.0)		11 (0.9)	9 (0.4)	
**24. On average, what is your frequency of masturbation-self-stimulation without a partner? (%)**				<.001			<.001
Daily	256 (7.5)	77 (3.5)	179 (14.5)		121 (9.7)	133 (6.6)	
Monthly	1025 (29.9)	726 (33.4)	297 (24.0)		374 (29.9)	647 (32.1)	
More than once a day	28 (0.8)	10 (0.5)	18 (1.5)		12 (1.0)	16 (0.8)	
Never	803 (23.5)	619 (28.5)	181 (14.6)		252 (20.1)	542 (26.9)	
Weekly	1136 (33.2)	626 (28.8)	508 (41.0)		475 (38.0)	660 (32.8)	
Missing	175 (5.1)	117 (5.4)	55 (4.4)		17 (1.4)	15 (0.7)	
**25. On average, what is your frequency of sex toys use (both alone or with a partner)? (%)**				<.001			.03
Daily	34 (1.0)	28 (1.3)	6 (0.5)		12 (1.0)	22 (1.1)	
Monthly	630 (18.4)	453 (20.8)	175 (14.1)		215 (17.2)	414 (20.6)	
More than once a day	5 (0.1)	3 (0.1)	2 (0.2)		3 (0.2)	2 (0.1)	
Never	2186 (63.9)	1281 (58.9)	900 (72.7)		868 (69.4)	1303 (64.7)	
Weekly	400 (11.7)	297 (13.7)	103 (8.3)		140 (11.2)	260 (12.9)	
Missing	168 (4.9)	113 (5.2)	52 (4.2)		13 (1.0)	12 (0.6)	
**26. On average, what is your frequency of sexual intercourse? (%)**				<.001			<.001
Daily	156 (4.6)	91 (4.2)	63 (5.1)		14 (1.1)	141 (7.0)	
Monthly	821 (24.0)	504 (23.2)	315 (25.4)		498 (39.8)	322 (16.0)	
More than once a day	16 (0.5)	13 (0.6)	3 (0.2)		2 (0.2)	14 (0.7)	
Never	462 (13.5)	335 (15.4)	127 (10.3)		368 (29.4)	82 (4.1)	
Weekly	1784 (52.1)	1111 (51.1)	670 (54.1)		344 (27.5)	1440 (71.5)	
Missing	184 (5.4)	121 (5.6)	60 (4.8)		25 (2.0)	14 (0.7)	
**27. On average, what is your frequency of orgasms with a partner? (%)**				<.001			<.001
10%-40% of the sexual activities	384 (11.2)	307 (14.1)	76 (6.1)		183 (14.6)	201 (10.0)	
41%-80% of the sexual activities	642 (18.8)	481 (22.1)	158 (12.8)		222 (17.7)	419 (20.8)	
Greater than 80% of the sexual activities	1656 (48.4)	830 (38.2)	823 (66.5)		410 (32.8)	1245 (61.8)	
Less than 10% of the sexual activities	274 (8.0)	234 (10.8)	40 (3.2)		190 (15.2)	81 (4.0)	
Never	269 (7.9)	191 (8.8)	78 (6.3)		215 (17.2)	50 (2.5)	
Missing	198 (5.8)	132 (6.1)	63 (5.1)		31 (2.5)	17 (0.8)	
**28. At what age do you think the individuals typically have their first sexual intercourse? (%)**				<.001			.8
<12	55 (1.6)	39 (1.8)	16 (1.3)		22 (1.8)	33 (1.6)	
>25	1 (0.0)	1 (0.0)	0 (0.0)		0 (0.0)	1 (0.0)	
12-15	1023 (29.9)	716 (32.9)	306 (24.7)		389 (31.1)	628 (31.2)	
13-15	171 (5.0)	65 (3.0)	106 (8.6)		57 (4.6)	113 (5.6)	
16-19	1903 (55.6)	1173 (53.9)	724 (58.5)		729 (58.3)	1163 (57.8)	
20-25	76 (2.2)	51 (2.3)	25 (2.0)		30 (2.4)	45 (2.2)	
Missing	194 (5.7)	130 (6.0)	61 (4.9)		24 (1.9)	30 (1.5)	
**29. What do you think the most common flaccid (soft) penile length is? (%)**				.07			.1
12.1 cm (4.8 in) to 16 cm (6.2 in)	99 (2.9)	66 (3.0)	32 (2.6)		38 (3.0)	60 (3.0)	
16.1 cm (6.3 in) or more	14 (0.4)	9 (0.4)	5 (0.4)		4 (0.3)	10 (0.5)	
4 cm (1.5 in) to 8 cm (3.1 in)	2004 (58.5)	1246 (57.3)	754 (60.9)		749 (59.9)	1244 (61.8)	
8.1 cm (3.2 in) to 12 cm (4.7 in)	933 (27.3)	596 (27.4)	337 (27.2)		355 (28.4)	575 (28.6)	
Less than 4 cm (1.5 in)	146 (4.3)	106 (4.9)	38 (3.1)		71 (5.7)	73 (3.6)	
Missing	227 (6.6)	152 (7.0)	72 (5.8)		34 (2.7)	51 (2.5)	
**30. What do you think the most common erect (hard) penile length is? (%)**				<.001			.008
12.1 cm (4.8 in) to 16 cm (6.2 in)	2015 (58.9)	1205 (55.4)	807 (65.2)		768 (61.4)	1239 (61.5)	
16.1 cm (6.3 in) or more	296 (8.6)	134 (6.2)	162 (13.1)		96 (7.7)	200 (9.9)	
4 cm (1.5 in) to 8 cm (3.1 in)	101 (3.0)	81 (3.7)	19 (1.5)		52 (4.2)	48 (2.4)	
8.1 cm (3.2 in) to 12 cm (4.7 in)	789 (23.0)	605 (27.8)	182 (14.7)		305 (24.4)	476 (23.6)	
Less than 4 cm (1.5 in)	5 (0.1)	5 (0.2)	0 (0.0)		0 (0.0)	5 (0.2)	
Missing	217 (6.3)	145 (6.7)	68 (5.5)		30 (2.4)	45 (2.2)	
**31. How long, after penetration, do you think it commonly takes a man to ejaculate/climax/cum? (%)**				<.001			<.001
<1 min	50 (1.5)	38 (1.7)	12 (1.0)		25 (2.0)	23 (1.1)	
>30 min	35 (1.0)	19 (0.9)	16 (1.3)		10 (0.8)	25 (1.2)	
1-2 min	235 (6.9)	178 (8.2)	56 (4.5)		113 (9.0)	119 (5.9)	
10.1-15 min	460 (13.4)	278 (12.8)	180 (14.5)		147 (11.8)	310 (15.4)	
15.1-20 min	233 (6.8)	145 (6.7)	88 (7.1)		74 (5.9)	159 (7.9)	
2.1-5 min	1017 (29.7)	669 (30.8)	345 (27.9)		427 (34.1)	586 (29.1)	
20.1-25 min	72 (2.1)	43 (2.0)	29 (2.3)		23 (1.8)	49 (2.4)	
25.1-30 min	50 (1.5)	35 (1.6)	15 (1.2)		17 (1.4)	33 (1.6)	
5.1-10 min	1062 (31.0)	630 (29.0)	431 (34.8)		386 (30.9)	671 (33.3)	
Missing	209 (6.1)	140 (6.4)	66 (5.3)		29 (2.3)	38 (1.9)	
**32. In your opinion what is the average volume of an ejaculation (cum)? (%)**				.07			.009
1.3 to 2.5 mL (1/4 to 1/2 teaspoon)	1077 (31.5)	691 (31.8)	383 (30.9)		431 (34.5)	638 (31.7)	
2.6-3.7 mL (1/2 to 3/4 teaspoon)	803 (23.5)	487 (22.4)	315 (25.4)		287 (22.9)	513 (25.5)	
3.8-4.9 mL (3/4 to 1 teaspoon)	689 (20.1)	424 (19.5)	264 (21.3)		263 (21.0)	426 (21.2)	
Greater than 4.9 mL (1 teaspoon)	477 (13.9)	317 (14.6)	158 (12.8)		160 (12.8)	312 (15.5)	
Less than 1.2 mL (less than 1/4 teaspoon)	150 (4.4)	103 (4.7)	47 (3.8)		69 (5.5)	80 (4.0)	
Missing	227 (6.6)	153 (7.0)	71 (5.7)		41 (3.3)	44 (2.2)	
**33. How long after ejaculation/climax, do you think it commonly takes a man to develop another erection for sexual activity? (%)**				.007			.6
<15 min	323 (9.4)	183 (8.4)	139 (11.2)		110 (8.8)	212 (10.5)	
>120 min	228 (6.7)	156 (7.2)	72 (5.8)		92 (7.4)	136 (6.8)	
15-30 min	1192 (34.8)	732 (33.7)	458 (37.0)		458 (36.6)	726 (36.1)	
31-60 min	1014 (29.6)	659 (30.3)	352 (28.4)		392 (31.3)	614 (30.5)	
61-120 min	448 (13.1)	299 (13.7)	149 (12.0)		166 (13.3)	281 (14.0)	
Missing	218 (6.4)	146 (6.7)	68 (5.5)		33 (2.6)	44 (2.1)	
**34. How many sexual partners, on average, do you think a man has in his lifetime? (%)**				<.001			<.001
<5	248 (7.2)	132 (6.1)	116 (9.4)		75 (6.0)	171 (8.5)	
>50	230 (6.7)	162 (7.4)	68 (5.5)		106 (8.5)	122 (6.1)	
11-20	1004 (29.3)	669 (30.8)	332 (26.8)		386 (30.9)	615 (30.6)	
21-50	628 (18.3)	454 (20.9)	173 (14.0)		241 (19.3)	383 (19.0)	
5-10	1001 (29.2)	592 (27.2)	406 (32.8)		359 (28.7)	636 (31.6)	
Missing	312 (9.1)	166 (7.6)	143 (11.6)		84 (6.7)	86 (4.3)	
**35. How frequently, on average, do you think a man typically uses pornographic content? (%)**				<.003			.3
Daily	829 (24.2)	552 (25.4)	276 (22.3)		336 (26.9)	490 (24.3)	
Monthly	647 (18.9)	383 (17.6)	263 (21.2)		235 (18.8)	409 (20.3)	
Never	113 (3.3)	83 (3.8)	30 (2.4)		46 (3.7)	65 (3.2)	
Weekly	1625 (47.5)	1016 (46.7)	605 (48.9)		604 (48.3)	1010 (50.2)	
Missing	209 (6.1)	141 (6.5)	64 (5.2)		30 (2.4)	39 (1.9)	
**36. How frequently, on average, do you think a man engages in sexual intercourse? (%)**				<.001			.2
Daily	320 (9.3)	258 (11.9)	60 (4.8)		124 (9.9)	194 (9.6)	
Monthly	558 (16.3)	291 (13.4)	266 (21.5)		235 (18.8)	320 (15.9)	
Never	13 (0.4)	11 (0.5)	2 (0.2)		4 (0.3)	8 (0.4)	
Weekly	2323 (67.9)	1476 (67.9)	843 (68.1)		858 (68.6)	1454 (72.2)	
Missing	209 (6.1)	139 (6.4)	67 (5.4)		30 (2.4)	37 (1.8)	
**37. How frequently, on average, do you think a man engages in masturbation (non-partnered self-stimulation)? (%)**				<.001			.05
Daily	1079 (31.5)	778 (35.8)	299 (24.2)		417 (33.3)	656 (32.6)	
Monthly	322 (9.4)	154 (7.1)	167 (13.5)		102 (8.2)	218 (10.8)	
Never	75 (2.2)	62 (2.9)	13 (1.1)		29 (2.3)	45 (2.2)	
Weekly	1735 (50.7)	1037 (47.7)	694 (56.1)		669 (53.5)	1059 (52.6)	
Missing	212 (6.2)	144 (6.6)	65 (5.3)		34 (2.7)	35 (1.7)	
**38. How frequently, on average, do you think a man uses a sex toy during masturbation (non-partnered self-stimulation)? (%)**				<.001			.06
Daily	44 (1.3)	33 (1.5)	11 (0.9)		18 (1.4)	25 (1.2)	
Monthly	1055 (30.8)	622 (28.6)	431 (34.8)		377 (30.1)	673 (33.4)	
Never	1738 (50.8)	1116 (51.3)	618 (49.9)		695 (55.6)	1036 (51.5)	
Weekly	354 (10.3)	250 (11.5)	104 (8.4)		121 (9.7)	229 (11.4)	
Missing	232 (6.8)	154 (7.1)	74 (6.0)		40 (3.2)	50 (2.5)	
**39. How frequently, on average, do you think a man uses a sex toy during partnered sex? (%)**				.2			.003
Daily	29 (0.8)	22 (1.0)	7 (0.6)		12 (1.0)	16 (0.8)	
Monthly	1705 (49.8)	1070 (49.2)	629 (50.8)		619 (49.5)	1077 (53.5)	
Never	1012 (29.6)	631 (29.0)	381 (30.8)		429 (34.3)	577 (28.7)	
Weekly	433 (12.6)	290 (13.3)	143 (11.6)		146 (11.7)	286 (14.2)	
Missing	244 (7.1)	162 (7.4)	78 (6.3)		45 (3.6)	57 (2.8)	
**40. How frequently, on average, do you think a man thinks about sex? (%)**				<.001			.4
Daily	1561 (45.6)	974 (44.8)	584 (47.2)		593 (47.4)	954 (47.4)	
From 5 to 10 times a day	324 (9.5)	242 (11.1)	80 (6.5)		133 (10.6)	190 (9.4)	
Monthly	6 (0.2)	4 (0.2)	2 (0.2)		2 (0.2)	4 (0.2)	
More than 10 times a day	410 (12.0)	258 (11.9)	151 (12.2)		146 (11.7)	263 (13.1)	
Never	7 (0.2)	5 (0.2)	2 (0.2)		1 (0.1)	6 (0.3)	
Up to 5 times a day	694 (20.3)	444 (20.4)	250 (20.2)		260 (20.8)	433 (21.5)	
Weekly	216 (6.3)	108 (5.0)	107 (8.6)		84 (6.7)	130 (6.5)	
Missing	205 (6.0)	140 (6.4)	62 (5.0)		32 (2.6)	33 (1.6)	
**41. How many sexual partners, on average, do you think a woman has in her lifetime? (%)**				<.001			.06
<5	541 (15.8)	363 (16.7)	176 (14.2)		205 (16.4)	331 (16.4)	
>50	48 (1.4)	19 (0.9)	29 (2.3)		16 (1.3)	32 (1.6)	
11-20	831 (24.3)	510 (23.4)	320 (25.8)		302 (24.1)	527 (26.2)	
21-50	293 (8.6)	184 (8.5)	109 (8.8)		108 (8.6)	183 (9.1)	
5-10	1393 (40.7)	924 (42.5)	465 (37.6)		534 (42.7)	850 (42.2)	
Missing	317 (9.3)	175 (8.0)	139 (11.2)		86 (6.9)	90 (4.5)	
**42. How long, on average, do you think it takes a woman to reach orgasm (cums) during sexual stimulation? (%)**				.001			.02
<2 min	18 (0.5)	16 (0.7)	2 (0.2)		5 (0.4)	12 (0.6)	
>30 min	184 (5.4)	119 (5.5)	65 (5.3)		91 (7.3)	93 (4.6)	
10.1-15 min	808 (23.6)	491 (22.6)	315 (25.4)		305 (24.3)	496 (24.6)	
15.1-20 min	607 (17.7)	386 (17.7)	220 (17.8)		243 (19.3)	363 (18.0)	
2-5 min	306 (8.9)	219 (10.1)	86 (6.9)		112 (9.01)	191 (9.5)	
20.1-25 min	303 (8.9)	196 (9.0)	107 (8.6)		107 (8.6)	196 (9.7)	
25.1-30 min	210 (6.1)	134 (6.2)	76 (6.1)		70 (5.6)	139 (6.9)	
5.1-10 min	770 (22.5)	469 (21.6)	298 (24.1)		282 (22.5)	483 (24.0)	
Missing	217 (6.3)	145 (6.7)	69 (5.6)		36 (2.9)	40 (2.0)	
**43. How frequently, on average, do you think a woman typically uses pornographic content? (%)**				1			.3
Daily	56 (1.6)	28 (1.3)	28 (2.3)		23 (1.8)	33 (1.6)	
Monthly	1854 (54.2)	1225 (56.3)	626 (50.6)		707 (56.5)	1138 (56.4)	
Never	539 (15.7)	336 (15.4)	201 (16.2)		219 (17.5)	317 (15.6)	
Weekly	747 (21.8)	438 (20.1)	308 (24.9)		265 (21.1)	476 (23.6)	
Missing	227 (6.6)	148 (6.8)	75 (6.1)		37 (3.0)	49 (2.4)	
**44. How frequently, on average, do you think a woman engages in sexual intercourse? (%)**				.3			<.001
Daily	123 (3.6)	73 (3.4)	49 (4.0)		42 (3.4)	81 (4.0)	
Monthly	847 (24.7)	527 (24.1)	320 (25.8)		363 (29.0)	479 (23.8)	
Never	16 (0.5)	12 (0.6)	4 (0.3)		3 (0.2)	13 (0.6)	
Weekly	2217 (64.8)	1415 (65.1)	796 (64.3)		802 (64.1)	1402 (69.6)	
Missing	220 (6.4)	148 (6.8)	69 (5.6)		41 (3.3)	38 (1.9)	
**45. How frequently, on average, do you think a woman engages in masturbation (non-partnered self-stimulation)? (%)**				.7			.7
Daily	193 (5.6)	119 (5.5)	74 (6.0)		78 (6.2)	113 (5.6)	
Monthly	1099 (32.1)	694 (31.8)	404 (32.6)		419 (33.5)	674 (33.5)	
Never	164 (4.8)	100 (4.6)	64 (5.2)		62 (5.0)	101 (5.0)	
Weekly	1740 (50.8)	1113 (51.2)	622 (50.2)		654 (52.3)	1077 (53.5)	
Missing	227 (6.6)	149 (6.9)	74 (6.0)		38 (3.0)	48 (2.4)	
**46. How frequently, on average, do you think a woman uses a sex toy during masturbation (non-partnered self-stimulation)? (%)**				.8			.2
Daily	125 (3.6)	78 (3.6)	47 (3.8)		56 (4.5)	68 (3.4)	
Monthly	1424 (41.6)	899 (41.3)	523 (42.2)		544 (43.5)	873 (43.4)	
Never	274 (8.0)	171 (7.9)	102 (8.2)		116 (9.3)	157 (7.8)	
Weekly	1368 (40.0)	873 (40.1)	491 (39.7)		496 (39.6)	864 (42.9)	
Missing	232 (6.8)	154 (7.1)	75 (6.1)		39 (3.1)	51 (2.5)	
**47. How frequently, on average, do you think a woman uses a sex toy during partnered sex? (%)**				.7			<.001
Daily	36 (1.1)	25 (1.1)	11 (0.9)		16 (1.3)	20 (1.0)	
Monthly	1871 (54.6)	1187 (54.5)	679 (54.8)		680 (54.3)	1183 (58.8)	
Never	680 (19.9)	421 (19.4)	258 (20.8)		306 (24.5)	369 (18.3)	
Weekly	606 (17.7)	393 (18.1)	212 (17.1)		208 (16.6)	395 (19.6)	
Missing	230 (6.7)	149 (6.9)	78 (6.3)		41 (3.3)	46 (2.3)	
**48. How frequently, on average, do you think a woman thinks about sex? (%)**				<.001			.03
Daily	1234 (36.1)	748 (34.4)	483 (39.0)		451 (36.1)	777 (38.6)	
From 5 to 10 times a day	67 (2.0)	40 (1.8)	27 (2.2)		22 (1.8)	45 (2.2)	
Monthly	301 (8.8)	175 (8.0)	126 (10.2)		124 (9.9)	175 (8.7)	
More than 10 times a day	59 (1.7)	24 (1.1)	35 (2.8)		16 (1.3)	42 (2.1)	
Never	17 (0.5)	15 (0.7)	2 (0.2)		7 (0.6)	10 (0.5)	
Up to 5 times a day	272 (7.9)	175 (8.0)	96 (7.8)		90 (7.2)	182 (9.0)	
Weekly	1260 (36.8)	858 (39.4)	399 (32.2)		506 (40.4)	745 (37.0)	
Missing	213 (6.2)	140 (6.4)	70 (5.7)		35 (2.8)	37 (1.8)	

### Language adaptation and validation

To ensure cross-cultural appropriateness and linguistic accuracy, translations underwent a bidirectional process: from English to each target language, then back to English for verification of meaning preservation. Thus, the English version underwent thorough translation and back-translation procedures across five languages: Italian, American English, Portuguese (Brazilian), Spanish, and Japanese. This process was coordinated across participating centers to maintain conceptual and linguistic equivalence.

### Statistical analysis

We conducted the analyses over two primary stages. The first stage focused on a descriptive analysis of findings according to gender assigned at birth, where respondents were grouped into females vs males (item 2). Of 48 items, for the specific purpose of this preliminary report, items #1, 2, 3, 4, 5, 6, 7, 8, 9, 14, 16, 18, 20, 22, 23, 25, 26, 27, 28, 29, 30, 31, 40, 42, and 48 have been discussed. The second stage examined findings on sexual satisfaction, where the cohort was stratified by sexual satisfaction status (satisfied vs not satisfied; item 22). Thus, for continuous variables, we reported medians with interquartile ranges (IQR), while categorical variables were presented as frequencies and proportions. Between-group comparisons were conducted using the Mann–Whitney test for continuous variables and Chi-square test for categorical variables. The analyses were conducted using R (2019), a language and environment for statistical computing (R Foundation for Statistical Computing).

### Ethical approval

The study protocol was approved by the institutional review boards of all participating centers. Informed consent was obtained from all respondents before questionnaire completion, regardless of distribution method. We maintained participant confidentiality and anonymity throughout the study process. No financial or other incentives were provided for participation.

## Results

Current findings represent the results of the descriptive analyses obtained using data from the YRC ISSM 48-item questionnaire only.

Table 1 depicts the descriptive statistics of the whole cohort, along with a further dichotomization according to genders assigned at birth and sexual satisfaction.

Overall, median (IQR) age was 39 (30-50) years. Of all, 2175 (63.5%) and 1238 (36.2%) reported their gender assigned at birth to be females and males, respectively. A large proportion of respondents had high educational levels, with 1325 (38.7%) holding post-graduate degrees and 1212 (35.4%) having a College-University degree, respectively. Most respondents were married, with 1686 (49.3%) in this category, while 764 (22.3%) were single persons. The overall relationship duration was 10 (5-20) years. Concerning sexual activity, 2852 (83.3%) respondents indicated they were sexually active over the last 12 months from questionnaire completion. Median age at first sexual intercourse was 18 (16-19) years, and the median number of lifetime sexual partners was 5 (3-11). Median age at first exposure to pornography was 14 (12-16) years. Of all, 1577 (46.1%) respondents claimed they never used pornography, while 834 (24.4%) and 181 (5.3%) reported using it on a monthly or daily basis, respectively. Satisfaction levels regarding sex life were reported as satisfactory by 1358 (39.7%) and very satisfactory by 655 (19.1%) respondents, respectively. In terms of sexual intercourse frequency, 1784 (52.1%) respondents engaged in sexual activities weekly. Of all, 1292 (37.7%) respondents agreed and 944 (27.6%) strongly agreed that penetration was important during sexual intercourse. Overall, 1656 (48.4%) achieved a satisfactory orgasm more than 80% of the time they engaged in sexual intercourse. Overall, 2186 (63.9%) respondents declared they had never used a sex toy (either alone or with a partner) (Table 1).

### Sexual orientation and identity distribution

Regarding sexual orientation, most respondents, 3092 (90%), identified themselves as heterosexual. When analyzed by gender assigned at birth, heterosexual orientation was predominant with 2056 (94.5%) females reporting attraction toward men and 1036 (83.7%) males reporting attraction toward women, respectively. A small proportion identified themselves as pansexual 46 (1.3%) or bisexual 11 (0.3%), respectively. Asexuality was reported by 13 (0.4%) respondents, predominantly among females 12 (0.6%). Moreover, 9 (0.3%) respondents, all females, indicated uncertainty about their sexual orientation, while missing data accounted for 10 (0.3%) respondents, thus displaying a possible uncertainty about sexual orientation. The distribution of sexual orientation showed significant differences between genders assigned at birth (*P* < .001), while no significant association was found with sexual satisfaction (*P* = .2) (Table 1).

### Demographic and religious characteristics

A wide range of religious beliefs were represented in the sample, with 1350 (39.4%) respondents identified as Christian-Catholic, as the most common religion. Regarding religious influence on sexual activity, 638 (18.6%) respondents agreed and 57 (1.7%) strongly agreed that their religious beliefs have impacted their sexual decisions. Conversely, 1167 (34.1%) disagreed and 763 (22.3%) strongly disagreed with this statement, and 722 (21.1%) remained neutral. Accordingly, a significant difference was observed between genders, with females reporting higher rates of agreement (440 (20.2%)) compared to males (196 (15.8%)) (*P* < .001). Notably, males showed a higher rate of strong disagreement (358 (28.9%)) compared to females (404 (18.6%)). Missing data accounted for 76 (2.2%) responses, equally distributed between females 47 (2.2%) and males 27 (2.2%), Table 1.

### Sexual norms and perceptions

Regarding sexual norms perception, 1903 (55.6%) respondents believed that individuals typically have their first sexual intercourse between 16 and 19 years of age. Missing responses regarding the perceived typical age of first sexual intercourse accounted for 194 (5.7%) of respondents, with a higher proportion among females compared to males 130 (6.0%) vs 61 (4.9%). This item showed one of the higher rates of missing data throughout the questionnaire. When evaluating penile length, 2004 (58.5%) respondents assumed the most common flaccid penile length to be between 4 cm (1.5 in) and 8 cm (3.1 in), while 2015 (58.9%) respondents believed the most common erect penile length to be between 12.1 cm (4.8 in) and 16 cm (6.2 in), respectively. Overall, 1062 (31.0%) respondents estimated the average time for a man to reach an orgasm to be 5.1-10 min, while 1017 (29.7%) estimated it to be 2.1-5 min. Conversely, 808 (23.6%) respondents believed a woman requires an average of 10.1-15 min to get orgasmic, while 770 (22.5%) estimated 5.1-10 min, respectively (Table 1).

### Sexual thoughts frequency

In total, 1561 (45.6%) and 1234 (36.1%) respondents answered that on average a man and a woman think about sex daily, respectively. Missing responses regarding the perceived frequency of sexual thoughts showed similar patterns between genders. For male sexual thoughts frequency, 205 (6.0%) responses were missing, with a higher proportion among females 140 (6.4%) compared to males 62 (5.0%). Similarly, for female sexual thoughts frequency, 213 (6.2%) responses were missing, again with a higher proportion among female respondents 140 (6.4%) compared to male respondents 70 (5.7%). The consistent pattern of missing data across both questions suggests similar levels of response reluctance for questions about perceived sexual thought frequency, regardless of the gender being assessed (Table 1).

### Gender-based analysis

As detailed, Table 1 also presents the most notable distinctions observed when stratifying the entire cohort according to the gender assigned at birth (females vs males). Females and males were similar in median IQR age 39 (30-50) vs 40 (30-52) years. Both groups exhibited similar rates regarding gender identity; among those assigned males at birth, 1209 (97.7%) still identified as males, and among those assigned females at birth, 2119 (97.4%) still identified as females. Females encountered pornography at an older median age (15 (12-17) vs 13 (12-15), *P* < .001) and engaged in sexual intercourse for the first time at an older median age (18 (16-20) vs 17 (16-19), *P* < .001). Females indicated a lower number of lifetime partners (5 (2-10) vs 7 (3-16), *P* < .001) compared to males. Instances of coerced, non-consensual sexual activity were more prevalent among females (543 (25%) vs 144 (11.6%), *P* < .001). Females exhibited higher rates of weekly sex toy usage during masturbation or partnered sexual activity (297 (13.7%) vs 103 (8.3%), *P* < .00). Regarding orgasm frequency during partnered sexual activity, females were less likely to experience orgasms than males. Only 830 (38.2%) females reported reaching orgasm more than 80% of the time during partnered sexual encounters, compared to 823 (66.5%) among men, *P* < .001. Concerning sexual norms perception, both females and males predominantly believed individuals engage in their first sexual intercourse between 16 and 19 years of age (1173 (53.9%) vs 724 (58.5%), *P* < .001). No differences between the groups were detected when estimating flaccid penile length. However, discrepancies emerged when assessing most common (“normal”) average erect penile length, with men more frequently reporting larger values than women [12.1 cm to 16 cm, in 807 (65.2%) vs 1205 (55.4%), *P* < .01 and >16.1 cm in 162 (13.1%) vs 134 (6.2%)]. Most respondents estimated the average ejaculation time for a man during penetrative sexual intercourse to be 2.1-5 min, 669 (30.8%) vs 345 (27.9%), *P* < .01 or 5.1-10 min, 630 (29%) vs 431 (34.8%), *P* < .001 for females and males, respectively. Conversely, when estimating the average time for a woman to get orgasmic during sexual intercourse, most respondents believed it to be within 10.1-15 min 491 (22.6%) vs 315 (25.4%), *P* = .001.

### Sexual satisfaction analysis

Upon assessing the entire cohort in terms of sexual satisfaction (not satisfied vs satisfied), Table 1 presents the most relevant distinctions. Notably, sexually satisfied respondents were slightly younger, median (IQR) age 38 (30-49) vs 41 (41-52) years, *P* < .001. No difference was observed between males and females or regarding sexual orientation. Respondents who were partnered were 517 (25.7) vs 186 (14.9%), *P* < .001 or married 1053 (52.3%) vs 530 (42.4%), *P* < .001 were more likely to report themselves as satisfied. A similar trend was found among respondents who had been sexually active within the previous 12 months were 1851 (92%) vs 888 (71%), *P* < .001 and respondents reporting weekly sexual intercourse exhibited greater satisfaction rates 1440 (71.5%) vs 344 (27.5%), *P* < .001. Interestingly, no variation in satisfaction rates were identified for religious beliefs, with 12 different religions being cited at least once. Respondents who claimed to achieve orgasm more than 80% of the time during sexual encounters showed greater satisfaction rates 1245 (61.8%) vs 410 (32.8%), *P* < .001, and those who disclosed experiences of coerced sexual activity without consent demonstrated higher rates of dissatisfaction 313 (25%) vs 372 (18.5%), *P* < .001. Moreover, relatively similar rates of satisfaction were seen for the different pornography consumption frequencies researched.

## Discussion

This pilot study of the ISSM YRC on an initial cohort of 3324 respondents from five countries and in five different languages revealed several key patterns in sexual norms and behaviors, with an interesting link to different aspects in the field of socio-cultural environment of sexuality and sexual function, which could eventually lead to develop an even more robust and comprehensive international survey.

First, respondents recruited showed significant gender (gender assigned at birth) differences in sexual experiences: females reported later age at first sexual intercourse and pornography exposure, lower numbers of lifetime partners, and notably lower orgasm frequency during partnered activities compared to males.

Second, perceptions of sexual norms varied by gender assigned at birth, particularly regarding erect penile length and time to orgasm. The descriptive analysis of these findings provided new perspectives toward sexual history, habits, and perceptions of sexual norms across the analyzed sample.

Third, although this pilot study, compared to an international survey, is absolutely not an investigation into the prevalence of sexual dysfunctions in the world population (unlike the famous Laumann’s study^10^) and it is also completely different in terms of respondents recruitment methods, type of survey and—and even more important—the socio-cultural contemporaneity of the events (years <2005 vs >2020), current findings align with Laumann’s Global Study of Sexual Attitudes and Behaviors, which demonstrated that predictors of sexual satisfaction remain largely consistent across different cultural contexts, despite varying with genders.^10^ Likewise, while acknowledging potential selection bias toward respondents interested in sexual health topics, our data revealed that nearly 60% of them reported being satisfied with their sex lives. This finding is particularly relevant as Flynn et al. have emphasized the significance of sexual health and satisfaction as essential components of overall quality of life.^11^ Furthermore, the analysis of current data identified several factors associated with sexual satisfaction, including higher frequency of sexual activity, having a committed partner, and achieving frequent orgasms (>80% of times). Once more, these findings are consistent previous data from Schoenfeld et al., who identified that the frequency of sexual intercourse was a predictor of both husbands’ and wives’ sexual satisfaction.^12^ Of note, none of all factors researched were a guarantee of satisfaction, nor an absolute certainty of dissatisfaction, supporting the complex nature of human sexuality and sexual satisfaction.

Fourth, the reported median ages at first sexual intercourse (18 years) and of first exposure to pornography (14 years) are noteworthy. In this context, given that exposure to pornography may often precede sexual debut, it is crucial for sexual education to address the potential impact of pornography on sexual attitudes and expectations at an early age. In this context, Rowland et al. importantly highlighted the potential association between a significant reliance on pornography consumption along with frequent masturbation with the increased likelihood of experiencing reduced sexual performance during partnered sexual activities and diminished satisfaction in relationships, particularly among more vulnerable subsets, which is important to take into consideration in the context of our survey.^13^ As such, the prevalence of pornography consumption in the sample was 48.5%, with its frequency being 24.4% of monthly, 18% weekly, 5.3% daily, and 0.8% more than once a day. Notably, higher pornography consumption rates were higher in males (*P* < .01). Our results warrant further investigation into the possible effects of pornography on sexual satisfaction and relationship dynamics.

Fifth, the analysis underscored the disparities in perceptions and attitudes toward sexual activity and norms across genders, religious beliefs, and education levels. Religious influence on sexual activity was reported by 18.6% of participants, though satisfaction rates were similar across religious beliefs. Hence, 20.3% of respondents either agree or strongly agree that their religious beliefs influenced their sexual activity, which may have implications for sexual education and counselling services tailored for individuals with specific religious backgrounds.^8^^,^^9^^,^^14^ Additionally, the high proportion of respondents with higher education degrees (74.1% holding College-University or post-graduate degrees) suggests that education level may play a role in shaping perceptions and attitudes toward sexual norms.^15^

Sixth, current findings regarding sexual orientation revealed a predominance of heterosexual orientation, with significant differences between genders assigned at birth. Notably, 94.5% of females reported attraction toward men and 83.7% of males reported attraction toward women. The lower proportion of respondents identifying as pansexual (1.3%), bisexual (0.3%), or asexual (0.4%) aligns with previous population-based studies, though our rates may be influenced by selection bias and cultural factors that affect sexual orientation disclosures. When segregating the whole cohort into genders assigned at birth (females vs males) several notable differences in sexual behavior, satisfaction, and perceptions of normalcy were also detected. In greater detail, there were differences found in the age of first exposure to pornography; indeed, males have been exposed significantly earlier as compared with females. Similarly, the age of first sexual intercourse also showed significant differences, since females encountered first sexual intercourse at later ages compared to males. This discrepancy may have implications for the content and timing of sexual education programs, which should consider gender-specific needs and experiences.^16^ In this regard, it has been widely stressed the fact that sexual education is an important factor in the context of first sex and later sexual health, and programs should continue to equip young people as they make immediate sexual behavior decisions and further sexual health-related decisions throughout their lifespan.^17^^,^^18^ Additionally, the results show that females reported a lower number of lifetime sexual partners and slightly lower sexual satisfaction rates than males. This disparity may be attributed to various speculative factors, thus including social and cultural norms, communication barriers, and differences in sexual preferences and expectations.^1^^,^^19^ These findings echo Haavio-Mannila and Kontula’s comparative research in Finland, which identified that women’s sexual satisfaction is influenced by factors such as sexual assertiveness, attitudes, and the importance placed on sexuality in life.^20^ Furthermore, our data show differences in orgasm frequency between genders, with fewer females reporting rates greater than 80% during sexual activities compared to males. This is in line with the scientific literature; for instance, by analyzing data from an internet questionnaire from 6151 men and women, Garcia et al. found that, regardless of sexual orientation, women have less predictable, more varied orgasm experiences.^21^ Our data regarding sexual satisfaction reveals that individuals in committed partnerships (either partnered or married) are more likely to report themselves as sexually satisfied. This can potentially indicate the impact of having a sexual partner, possibly favoring regular sexual activity. Supporting that, those who have been sexually active within the past year, experience heightened levels of satisfaction, and higher sexual frequencies were associated with higher satisfaction. This supports the idea that emotionally fulfilling, stable relationships can contribute positively to one’s sexual satisfaction. However, it is important to note that the Global Better Sex Survey (GBSS) by Mulhall et al. had highlighted erectile function and the impact of erectile dysfunction on various aspects of the sexual experience as the foremost concerns for male respondents in terms of sexual satisfaction.^1^

Of note, the elevated prevalence of coerced, non-consensual sexual activity among females is a concerning issue that warrants attention and action. Extensive literature documents the detrimental effects of non-consensual sex on its victims, encompassing both physical and emotional harm. Prevalence rates have been reported to reach as high as 40% over the lifetimes of American women.^22^^,^^23^ This terminology is used in the literature to encompass both violent and non-violent coercion, consistently underscoring its higher occurrence among women.^24^ In fact, numerous reports highlight significantly high rates of non-consensual sexual activity among females. For instance, a study conducted in Benin and Nigeria revealed that approximately one in five respondents reported experiencing non-consensual sexual activity, with a majority of perpetrators being the victims’ present partners, followed by husbands (22.2%).^24^ Maintaining scientific vigilance regarding these rates and regularly collecting such data can drive more effective initiatives aimed at reducing this pervasive global burden. Together, our findings reinforce the notion that perceptions of sexual normality are shaped by complex biopsychosocial factors, as evidenced by the observed gender-based discrepancies in perceived penile size and time to orgasm. Given that distorted or unrealistic norms may contribute to individual distress, and considering recent societal changes, further studies with broader and more diverse samples are warranted to explore the extent to which these perceived norms translate into psychological burden or maladaptive sexual behavior. By acknowledging these socio-cultural dynamics, Sexual Medicine practitioners can refine counselling approaches and promote more individualized treatment plans.

### Strengths and limitations

Although it is certainly largely interesting and covers a considerable area in terms of socio-cultural aspects and related to sex assigned at birth, the findings of this pilot survey from ISSM YRC are certainly not devoid of limitations, and caution should be taken on the overall interpretation. First, the cross-sectional design precludes causal inferences, and the self-reported and multimodal nature of the data may be subjected to recall bias and social desirability bias. Second, despite among the few surveys to report real-life contemporary data from a number of countries around the world, the sample may not be actually representative of the broader population, as it relies on voluntary participation and may be influenced by selection biases. Thereof, despite collecting data across five countries, the uneven distribution of respondents prevented reliable cross-cultural comparisons, highlighting the need for future studies with stratified sampling strategies to ensure balanced representation across different countries and cultural groups. In this regard, it is imperative to develop adequate surveys and conduct future research that employs a massive and strategic distribution of questionnaires/instruments, aiming to enhance the generalizability of the findings. Also, incorporating a longitudinal design would be highly beneficial to validate current observations, examine changes in sexual behaviors and attitudes, satisfaction, and perceptions over time, as well as exploring potential mediators and moderators of these relationships.

## Conclusions

This ISSM-supported pilot survey explored preliminary data on gender-based differences in terms of sexual behaviors, satisfaction, and perceptions of normality from a five-country, five-language contemporary real-world investigation. Our findings highlight significant differences between males and females in sexual experiences, satisfaction rates, and response patterns, as well as revealing the impact of religious beliefs and partnership status. As a preliminary investigation, this work establishes groundwork for expanded research in this field proving its feasibility.
